# Assessing the Appeal of Instagram Electronic Cigarette Refill Liquid Promotions and Warnings Among Young Adults: Mixed Methods Focus Group Study

**DOI:** 10.2196/15441

**Published:** 2019-11-25

**Authors:** Linnea I Laestadius, Kendall E Penndorf, Melissa Seidl, Young I Cho

**Affiliations:** 1 Zilber School of Public Health University of Wisconsin-Milwaukee Milwaukee, WI United States

**Keywords:** social media, vaping, tobacco, marketing

## Abstract

**Background:**

While marketing for electronic cigarette refill liquids (e-liquids) is widespread on Instagram, little is known about the post elements that create appeal among young adult Instagram users. Further information is needed to help shape regulatory strategies appropriate for social media.

**Objective:**

This study examined young adult Instagram user perceptions of actual e-liquid marketing posts and US Food and Drug Administration (FDA)–mandated nicotine addiction warning statements on Instagram.

**Methods:**

A series of 12 focus groups (n=69) were held with non–tobacco users, vapers, smokers, and dual users in Wisconsin between September and December 2018. Participants discussed the elements of posts that they found appealing or unappealing, in addition to completing a survey about each post and e-liquid. Focus group transcripts were analyzed by smoking status using a framework analysis approach.

**Results:**

Although willingness to try e-liquids was highest among nicotine users, focus group discussions indicated that Instagram posts promoting e-liquids held appeal for individuals across smoking statuses. The primary elements that created appeal were the perceived trustworthiness of the Instagram account, attractive design and flavor visuals, and promotion of flavors and nicotine levels that met personal preferences. Post appeal was reduced by references to vaping subcultures, indicators that the post creator did not take nicotine addiction seriously, and FDA-mandated nicotine warning statements. Non–tobacco users were particularly drawn to posts featuring nicotine-free e-liquids with attractive visual designs and flavors known from foods.

**Conclusions:**

Young adults consider a broad range of elements in assessing the appeal of e-liquid marketing on Instagram, with minor but notable distinctions by smoking status. Non–tobacco users are uniquely drawn to nicotine-free e-liquids and are more deterred by the FDA’s mandated nicotine addiction warning statements than those from other smoking statuses. This suggests that it may be possible to tailor policy interventions in a manner that helps to reduce novel uptake of vaping without significantly diminishing its potential harm-reduction benefits.

## Introduction

With the growing popularity of refillable electronic cigarettes (e-cigarettes) [1], marketing for the aerosolized electronic cigarette refill liquids (e-liquids) used with devices has become increasingly prevalent. Early estimates suggested that there were thousands of flavors of e-liquids available for sale online [2]. Considering the extremely large variety of e-liquids on the market, brands have sought to distinguish their products using creative flavors, names, label designs, and advertising visuals [3,4]. Many e-liquids are promoted on the visual social media platform Instagram, which is used by 71% of young adults aged 18 to 24 in the United States [5]. While Instagram bans paid advertising for tobacco products, both e-liquids and e-cigarettes are regularly promoted through posts made by vendors, brands, and sponsored users [3,6,7]. This content remains largely unregulated, except for US Food and Drug Administration (FDA) regulations requiring nicotine addiction warning statements to be placed on all visual advertising for e-liquids containing nicotine. The FDA has clarified that these provisions apply to advertising “communicated via mobile telephone, smartphone, microblog, social media Web site, or other communication tool” [8].

Despite the rapid growth of this content, little is known about the elements of promotional e-liquid posts that shape appeal among young adults. Analyses of Instagram post metadata suggest that e-cigarette posts focused on products receive more likes than explicitly promotional posts [7]. Experimental studies using artificial posts indicate that celebrity endorsements of e-cigarettes on Instagram are associated with greater intent to use e-cigarettes [9,10], although formal celebrity vaping promotions are not currently prevalent on Instagram [3,11]. Exposure to cartoons on e-liquid bottles has also been shown to be associated with a susceptibility to use e-cigarettes [12]. Studies of Instagram promotion from other fields suggest that high quality images and avoiding resemblances to traditional advertising are key to establishing appeal and authenticity [13,14]. There is also limited evidence on the ways in which FDA-mandated nicotine warnings might alter the appeal of posts. To date, only one study has considered these warnings on social media. Focusing on a sample of e-cigarette users, the study found that warnings on tweets from a fictional e-cigarette company reduced perceptions of healthiness but did not increase perceptions of harm or reduce willingness to try e-cigarettes [15].

To address current gaps in the literature and determine the post elements that shape appeal, this study examined perceptions of actual Instagram posts that promote e-liquids through a series of twelve focus groups with young adults. Evidence suggests that young adults are at higher risk of initiating e-cigarette use than youth [16], making this a particularly important population for study. Considering both the importance of e-cigarettes as a harm reduction tool for smokers and the challenges of growing uptake of e-cigarettes by nonsmokers [17], we also considered how perspectives differ by smoking status. Finally, we examined the impact of FDA-mandated warning statements about nicotine addiction. Study insights can help to determine priorities for the regulation of e-cigarette promotions on visual social media.

## Methods

### Summary

Young adults in the Milwaukee, Wisconsin area were recruited through ads on Facebook, Instagram, and Craigslist, as well as flyers distributed at local vape and tobacco stores, coffee shops, libraries, and one public and one private university. To be eligible, participants had to be aged 18-24 years old, speak English, and view their Instagram account at least once per week. Participants were screened by a graduate research assistant and those who met the above criteria were invited to join the study. Participants were assigned into focus groups based on their status as a smoker, vaper, dual user, or non–tobacco user. Former smokers who did not currently vape were excluded from enrollment. Participants were provided with a meal and a $50 gift card as an incentive. The Institutional Review Board at the University of Wisconsin-Milwaukee approved the research protocol.

### Data Collection

Overall, 12 focus groups were held between September and December 2018. Prior research suggests that most themes are identified in the first three focus groups, so we aimed for three groups per smoking status [18]. Focus groups were stratified by smoking status and sizes ranged from four to eight participants. Each focus group lasted approximately 90 minutes. A semistructured interview guide was developed to elicit perceptions of five real world Instagram posts promoting e-liquids. Posts were chosen to be representative of common themes, user types, and practices found in e-liquid promotions on Instagram [3]. Specifically, posts were chosen to reflect both vendors and individual users with commercial ties to brands. Posts were also chosen to reflect elements such as warning statements, flavor descriptions and visuals, cartoon illustrations, nicotine and nicotine-free e-liquids, and the presence of a person vaping. To protect the privacy of the accounts whose posts were included, Multimedia Appendix 1 includes a written description of posts rather than post reproductions. To capture greater variation in post elements and include more posts with the FDA nicotine warning statements, which became mandatory effective August 2018, three of the posts were switched out for new ones after the completion of the first focus group for each smoking status. Mandated warnings comprise 20% of the visual element of the post, and state: “WARNING: This product contains nicotine. Nicotine is an addictive chemical.”

Following informed consent, participants completed questionnaires on self-described demographics, tobacco use, and prior e-liquid marketing exposure. Participants were also presented with printed copies of each post. To provide participants with the full context needed to assess posts, these were paired with images of the user profile pages associated with posts (containing the user’s profile information and small versions of their three most recent Instagram posts). For each post, participants were asked to circle elements they found appealing or unappealing and complete a survey indicating if they would like, comment on, or repost the post, their willingness to purchase or try the e-liquid, and their perception of the e-liquid using a response scale, modeled after prior research on perceptions of e-cigarette ads [19] and based on the following word pairs: delicious/disgusting, enjoyable/unenjoyable, healthy/unhealthy, safe/dangerous, fun/boring, and cool/not cool. For each word pair, participants used a 5-point scale to indicate which word better captured their impressions (eg, fun would be a 5 and boring would be a 1). Led by a professional, independent moderator, each post was discussed one at a time, with participants prompted to explain elements that they found appealing or unappealing. All focus groups were audio-recorded.

### Data Analysis

First, quantitative data from the survey were analyzed across smoking status groups. Because each individual participant rated 5 Instagram images and therefore those 5 ratings are nonindependent, meaning that ratings are clustered within each individual (a total of 345 ratings by 69 participants), analyzing such nonindependent data as if they were independent can lead to biased estimates. To account for nonindependence, we employed mixed-effects models (mixed-effects logistic models for binary variables, and mixed-effects regression models for the 5-point Likert scale values) [20]. All statistical analyses were conducted using STATA 14 (StataCorp, College Station, Texas).

Following transcription of focus group discussions, qualitative data were analyzed using a framework analysis approach [21]. The first transcript from each smoking status was independently coded line-by-line by the lead (LL), second (KP), and third (MS) authors to allow for discussion of commonly found themes and the creation of an initial codebook. Themes were identified using an inductive process, with initial codes being grouped into higher level codes through team discussion [22]. Following this, the codebook was applied to all transcripts by the first author (LL) and second author (KP) using MAXQDA 2018 (VERBI Software, Berlin, Germany). All coding was discussed, with any disagreements resolved and updates to code definitions made as needed. Following coding, all data were charted by primary theme and smoking status into a spreadsheet matrix to facilitate analysis and interpretation. Additionally, memos were used to summarize and explore relations between themes, identify differences by smoking status, and highlight illustrative quotes. Memos took the form of multiple collaborative Word documents exploring different themes to assist with data immersion and the leap from “the concrete to the conceptual” [23]. The findings below capture themes related to the elements that shape the appeal of e-liquid marketing on Instagram. For brevity, smokers, vapers, and dual users together are collectively referred to as “nicotine users”.

## Results

### Overview

We conducted a total of 12 focus groups with 69 individuals; three focus groups each with vapers (n=20), dual users (n=18), smokers (n=12), and non–tobacco users (n=19). Overall, the sample was 58% male (n=40) and 41% female (n=28), with an average age of 21.10. One participant identified as nonbinary. See Table 1 for further participant demographics. A total of 45% (n=9) of vapers were former smokers, while 58% (n=7) of smokers reported having tried e-cigarettes at least once. Overall, 50% (n=9) of dual users and 83% (n=10) of smokers reported smoking cessation attempts in the past year. Post surveys indicated that about one third of participants would like the posts if they saw them on their Instagram feed. Only 7% (n=5) and 4% (n=3) of them would comment or repost, respectively. The group difference on these opinions was found not to be statistically significant. However, vapers were more likely to rate e-liquids “healthy” and “safe” than nonusers. All nicotine user statuses also expressed a greater willingness to try the e-liquids if offered by a close friend and to buy them for themselves. See Table 2 for full post ratings and Table 3 for full e-liquid ratings. In Table 3, means represent ratings from 1-5, with 5 representing the most favorable rating of the e-liquid. Standard errors were adjusted for clustering of the measures within respondents.

**Table 1:** Participant characteristics by smoking status.

Characteristics		Total (N=69)	Nonuser (n=19)	Cigarette smoker (n=12)	Vaper (n=20)	Dual user (n=18)
Age (mean, SD)		21.10 (1.91)	21.11 (2.08)	22.92 (1.31)	19.75 (1.21)	21.39 (1.65)
**Gender, n (%)**						
	Male	40 (58.0)	9 (47.4)	9 (75.0)	14 (70.0)	8 (44.4)
	Female	28 (40.6)	9 (47.4)	3 (25.0)	6 (30.0)	10 (55.6)
	Nonbinary	1 (1.4)	1 (5.2)	0 (0)	0 (0)	0 (0)
**Race/ethnicity, n (%)**						
	White	43 (62.3)	9 (47.4)	3 (25.0)	17 (85.0)	14 (77.8)
	African American/black	11 (15.9)	6 (31.6)	3 (25.0)	0 (0)	2 (11.1)
	Hispanic/Latinx	3 (4.4)	0 (0)	3 (25.0)	0 (0)	0 (0)
	Asian	6 (8.7)	1 (5.3)	2 (16.7)	1 (5.0)	2 (11.1)
	American Indian	1 (1.5)	1 (5.3)	0 (0)	0 (0)	0 (0)
	Biracial	5 (7.2)	2 (10.5)	1 (8.3)	2 (10.0)	0 (0)
**Education, n (%)^a^**						
	High school graduate	9 (13.2)	1 (5.6)	1 (8.3)	4 (20.0)	3 (16.7)
	Some college or technical training	47 (69.1)	11 (61.1)	8 (66.7)	16 (80.0)	12 (66.7)
	College graduate or more	12 (17.6)	6 (33.3)	3 (25.0)	0 (0)	3 (16.7)
**Seen/heard promos for e-cigs^b^/e-liquids^c^, n (%)^a^**						
	In stores	45 (66.2)	13 (72.2)	10 (83.3)	11 (55.0)	11 (61.1)
	TV	11 (16.2)	7 (38.9)	0 (0)	2 (10.0)	2 (11.1)
	Radio	2 (2.9)	1 (5.6)	1 (8.3)	0 (0)	0 (0)
	Instagram	40 (58.8)	12 (66.7)	6 (50.0)	12 (60.0)	10 (55.6)
	Other social media	40 (58.8)	13 (72.2)	5 (41.7)	11 (55.0)	11 (61.1)
	Website banner ads	20 (29.4)	7 (38.9)	1 (8.3)	8 (40.0)	4 (22.2)
	Billboards	7 (10.3)	2 (11.1)	2 (16.7)	2 (10.0)	1 (5.6)

^a^n=18. One nonuser did not list education or prior marketing exposure.

^b^e-cigs: electronic cigarettes.

^c^e-liquids: electronic cigarette refill liquids.

**Table 2:** Ratings of the posts by smoking status.

Ratings	Total, proportion (SD)	Nonuser, proportion (SD)	Cigarette smoker, proportion (SD)	*P* value^a^	Vaper, proportion (SD)	*P* value^a^	Dual user, proportion (SD)	*P* value^a^
Would like post	0.33 (0.04)	0.28 (0.06)	0.48 (0.10)	.06	0.33 (0.06)	.61	0.30 (0.07)	.93
Would comment on post	0.07 (0.02)	0.13 (0.06)	0.08 (0.04)	.75	0.05 (0.03)	.28	0.02 (0.02)	.11
Would repost	0.04 (0.01)	0.01 (0.01)	0.08 (0.05)	.13	0.02 (0.02)	.79	0.05 (0.03)	.27

^a^*P* values were obtained from mixed-effect models with nonusers as a reference group.

**Table 3:** Ratings of the electronic cigarette refill liquids by smoking status.

Ratings	Total, mean (SD)	Nonuser, mean (SD)	Cigarette smoker, mean (SD)	*P* value^a^	Vaper, mean (SD)	*P* value^a^	Dual user, mean (SD)	*P* value^a^
Delicious	3.21 (0.08)	3.03 (0.15)	3.17 (0.21)	.48	3.19 (0.13)	.45	3.41 (0.15)	.08
Enjoyable	3.31 (0.08)	3.10 (0.15)	3.27 (0.21)	.53	3.37 (0.16)	.23	3.48 (0.15)	.10
Healthy	2.51 (0.10)	1.95 (0.14)	2.51 (0.15)	.05	3.04 (0.19)	<.001	2.47 (0.20)	.04
Safe	2.85 (0.10)	2.37 (0.13)	2.90 (0.22)	.07	3.30 (0.21)	<.001	2.78 (0.19)	.11
Fun	3.44 (0.09)	3.13 (0.14)	3.64 (0.32)	.07	3.46 (0.16)	.17	3.58 (0.13)	.07
Cool	3.04 (0.12)	2.62 (0.20)	3.22 (0.35)	.09	3.14 (0.17)	.08	3.21 (0.21)	.05
Would try	3.61 (0.12)	2.80 (0.29)	3.54 (0.21)	.02	4.01 (0.12)	<.001	3.97 (0.21)	<.001
Would buy	2.15 (0.10)	1.52 (0.15)	2.24 (0.23)	.007	2.26 (0.16)	.003	2.57 (0.21)	<.001

^a^*P* values were obtained from mixed-effect models with nonusers as a reference group.

### Focus Group Discussions

#### Overview

Through framework analysis we identified the following themes in how young adults assess the appeal of e-liquid marketing posts on Instagram: (1) accounts must be trustworthy; (2) visuals are key to grabbing attention; (3) flavor mimicry, in which flavors resemble known foods and beverages, is favored; (4) nicotine levels should be clear and have options; (5) references to vaping culture repel users and nonusers alike; (6) marketing should be sensitive to nicotine addiction; and (7) warning statements reduce post appeal. Each of these and their subthemes are covered below, with discussion of any differences tied to smoking status. Illustrative quotes are found in Table 4.

#### Accounts Must be Trustworthy

Participants of all smoking statuses frequently reflected on the importance of the trustworthiness of the users who made the posts. Limited trust in a user, or the company they were promoting, diminished interest in trying a product due to both safety and ethical concerns. Without the ability to try a product in person, participants relied on proxy measures for quality. The number of followers, likes, and comments were frequently mentioned as a good sign that a poster was trustworthy (Table 4: Q1.1). Several participants were concerned about the ratios of followers to following and about fake followers, which caused trust to significantly diminish. Across all the groups, many participants found posts from individual Instagram users with brand/store sponsorships to be appealing and authentic and therefore more trustworthy than more explicitly commercial posts made directly by brands or stores (Table 4: Q1.2). The more the post felt like organic, unpaid content, the more trust increased. For many there was a sense of inherent trust in the posters, and sponsorship conferred some sense of the product having been vetted. One non–tobacco user felt that sponsored posts were particularly reliable because:

you’re not going to just like let yourself be sponsored by anything.

However, when sponsored users crossed the line into being “someone who just has sponsors for money,” their higher levels of appeal begin to diminish (Table 4: Q1.3).

**Table 4:** Selected quotes highlighting key themes in responses to e-liquid^a^ promotion posts.

Theme		Quote^b^
**Accounts must be trustworthy and authentic.**		
	1.1	*The more [followers] you have the more accountable you are to more people, so I trust something from someone who has a lot more followers than someone who has a lot less.* [V^c^ 47]
	1.2	*I just feel more trust for this post than I would someone that’s like purely trying to sell me something... It’s just more of a personal picture because it could be one of my friends posting a picture of what they’re smoking rather than like a graphic that someone obviously spent money for a designer [to create]…* [DU^d^ 26]
	1.3	*Like maybe he’s not a company, but he’s definitely like a promoter and someone is paying him to do this… it takes me out of the whole personal thing, it doesn’t feel as personal.* [DU 32)]
**Visuals are key to grabbing attention.**		
	2.1	*Regardless of the how good the flavors sound, you’re not going to look in the description unless you like the main picture...* [DU 20]
	2.2	*I’m not going to lie, I marked like it looked fun mostly because of the colors within it...* [NU^e^ 2]
	2.3	…*it’s easy to look at that and I think of the flavor the strawberry and imagining like how it kind of does make you think of gum and like you can easily imagine what a fruity minty gum tastes like and so, even though it’s not gum obviously, but it’s easy to image what that would be like tasting it and also I smoke menthol cigarettes so, the whole- the specific thing of like refreshing, menthol, and then ice cubes it’s like “yes, that’s- I like that, that’s up my alley”.* [CS^f^ 21]
**Flavor mimicry is favored.**		
	3.1	*If I didn’t use it, this would be the one that would make me want to, simply because I like candy and it’s something that I know I would like if it actually did imitate the taste or something like that, so I thought it was cool.* [NU 21]
	3.2	*Yeah, I mean I love coffee so it definitely stood out as intriguing, caught my attention.* [NU 26]
	3.3	*Because it doesn't have nicotine and it looks like a pie flavor so, I have smoked regular cigarettes and it’s not like you have these kinds of flavors. It’s just something that I may want to explore... It’s something that I might want to explore, you know, to try different flavors like in a smoking experience.* [CS 23]
**Nicotine levels should be clear and have options.**		
	4.1	*I hit mine just for the buzz. I don’t really see the point of hitting for a cloud [of vapor]… I wouldn’t buy it because I don’t really see the point.* [V 47]
	4.2	*I know the difference between lights and Marlboro lights and Marlboro reds and menthol and that kind of stuff but, if someone tells me like, “Okay, this vape has 21 milligrams of nicotine,” I have no idea what that amounts to....So, instead of just putting out numbers make it easier in a way to understand people that look at this as, this has more nicotine as compared to a normal one. This is something which has less.* [CS 27]
	4.3	*If I were to vape, I would probably start with something that doesn’t have nicotine and, how am I supposed to know, if it had like a label that’s probably what I’m going to reach for.* [NU 09]
**References to vaping culture repel users and nonusers alike**		
	5.1	*It makes me not want to buy it like at all... Yeah. I don't know, it’s just overly masculine. Yeah “be like us use this e-liquid” or whatever, just like some Chad. I don't know, I hate everything about this ad.* [V 31]
	5.2	*There’s no imagery of like vaping in movies and shit…you don’t see like a Swashbucklers, like a cool character, or like bad boy with a vape. It’s always a cigarette.* [CS 18]
	5.3	*None of [the posts] seem like they are at all geared towards quitting smoking, it’s just promoting the hobbyist side of vaping...I don’t know, if I were to go through the process of quitting something, I wouldn’t want to make it this glamorized thing of like “oh now I’m joining this cool vaping community.” I’d just be like “oh wow, I’m damaging my body every day and I want to try to damage my body a little bit less.” I don’t know if I would necessarily buy into that as “this is my new hobby now,” like it’s the same as if someone said that their hobby was going sober and not drinking alcohol...* [DU 20]
**Marketing should be sensitive to nicotine addiction**		
	6.1	*I feel like it’s representing the vape community poorly and makes it seem like we’re trying to market, or they’re trying to market, to young people and there are just a bunch of average people like us who vape, for whatever reason that we do, and aren’t trying to promote this negatively. So, I think it just reflects poorly.* [V 30]
	6.2	*The whole point of e-cigarettes is to help people use a healthier alternative to smoking and eventually quit nicotine or cigarettes all together, but this guy is treating it as a hobby which is like super lame. For example, like the zero milligrams and he even has a hashtag that says “stop smoking.” Like there’s really no point of him even putting this in his lungs if he’s smoking zero milligrams.* [CS 18]
	6.3	*You shouldn’t brag about being addicted to something that isn’t good for you. Like it’s- not negative it just doesn’t need to be there, like if there is like some alcohol company showing off their bottle, I guarantee they wouldn’t put ‘alcoholic’ as a hashtag.* [V 29]
**Warning statements reduce post appeal**		
	7.1	*It’s kind of like album artwork when they have the explicit sign on it and it’s really pretty album artwork it’s like – I get it, it has swearwords, why did you have to put that there? You just ruined the album artwork. And it’s kind of the same idea.* [V 40]
	7.2	*I already smoke cigarettes, there’s already nicotine in that so-(laughs).* [CS 4]
	7.3	*I don’t know, it’s like putting ‘warning’ on like a beer thing, “warning this beer will get you drunk,” like yeah, no shit, it will.* [V 31]

^a^e-liquid: electronic cigarette refill liquids.

^b^Quotes are identified by smoking status (V, DU, CS, or NU) and participant number.

^c^V: vaper.

^d^DU: dual user.

^e^NU: non–tobacco user.

^f^CS: cigarette smoker.

#### Visuals are Key to Grabbing Attention

All participant groups favored good visual design, defined largely by how well posts met conventions for “good Instagramming” (eg, not too many hashtags, not reposting identical images, use of aesthetically appealing filters), the clarity with which posts conveyed flavors and nicotine levels, and personal preferences for colors, visuals, and themes used in marketing. Given the Instagram ecosystem, in which users must choose to stop on a post and read it rather than continuing to scroll through their feed, some participants explicitly recognized the importance of initial post impression (Table 4: Q2.1).

Although thematic and aesthetic preferences were highly individual, with participants in the same group often strongly divided on the visual appeal of a post, most users favored fun and bright-colored posts (Table 4: Q2.2). Pop culture themes were also attractive to many. A post featuring an e-liquid with an anthropomorphic cartoon banana on the label and the caption “This flavor is B-A-N-A-N-A-S” was particularly popular and made several participants think of a song by pop artist Gwen Stefani. Overly edgy themes were more broadly disliked due to heightened risk perceptions and a sense of companies trying too hard to be cool. Visuals depicting flavors were almost universally appreciated when the foods aligned with their own taste preferences. Flavor visuals allowed participants, including non–tobacco users, to conjure up a vivid image of the flavor (Table 4: Q2.3).

#### Flavor Mimicry is Favored

As suggested above by the importance of flavor visuals, the flavors of the e-liquids being marketed were critical for determining appeal. Although individual flavor preferences were varied, which suggests that multiple flavor options are critical, most participants expressed liking flavors that mimicked tastes that they already knew and enjoyed in the context of real foods and beverages. Several non–tobacco users expressed strong interest in known flavors (Table 4: Q3.1, Q3.2). Specific named items like branded candies were particularly appealing because they were considered to be increasing the odds of flavors tasting good.

Unlike non–tobacco users, who mainly felt that flavors seemed appealing, vapers, dual users, and smokers had concerns with flavor fidelity and the extent to which flavors accurately captured the flavor they were intended to portray. Several e-liquids were deemed unappealing because the users felt that manufacturers probably failed to capture the flavor correctly. Vague e-liquid names and posts without clear flavor information also reduced post appeal. Many vapers and dual users felt that visuals were important for initial appeal, but flavor was what that ultimately shaped their perception of the e-liquids. As a result of this, several participants indicated that they would be willing to try e-liquids in the posts but would not outright buy them in case they disliked the flavor. For smokers who did not currently vape, e-liquid flavors presented a distinct point of interest because it was a way to branch out from the taste of tobacco. Some smokers indicated that they might entertain the idea of vaping because of the flavors depicted in the posts (Table 4: Q3.3)

#### Nicotine Levels Should be Clear and Have Options

Participants from all smoking statuses wanted posts to provide clear information about nicotine levels. Among nicotine users, there was a desire to know about the availability of higher nicotine levels than the ones depicted in the post. This was particularly the case for posts depicting bottles with 0 to 3 milligrams of nicotine, which many nicotine users found to be insufficient to meet their own needs (Table 4: Q4.1). Still, most vapers and dual users felt more inconvenienced than deterred by unappealing nicotine levels because they indicated that the e-liquid flavor would likely be sold in multiple nicotine levels if they looked it up online. Smokers seemed less aware of the variety of nicotine levels available and were not always clear on which level of nicotine would meet their needs (Table 4: Q4.2). Overall, nicotine users also had an appreciation for e-liquids offered in a variety of nicotine levels, with several noting that nicotine users could gradually taper their nicotine levels for health or cessation.

Non–nicotine users wanted to know which e-liquids were nicotine-free and considered clear labeling to be useful. Some suggested this could be like peanut-free or sugar-free labeling, which was partially out of concern for former smokers who they envisioned might want to vape but not use nicotine, while others appeared to consider their own potential use of e-liquids (Table 4: Q4.3). The presence of nicotine was seen as elevating the risks of the product, while non–nicotine products were more welcoming and less risky.

#### References to Vaping Culture Repels Users and Nonusers Alike

Participants felt that several of the Instagram posts captured images, themes, and language that were representative of vaping culture. The presence of these post elements reduced appeal among large numbers of participants across all smoking status groups. A post depicting a tattooed man in a baseball cap vaping and holding an e-liquid elicited particularly negative responses. The frequent use of vape culture hashtags referencing women (eg, #girlswhovape, #dripgirls), despite the lack of depiction of women in posts, was particularly unappealing to female participants. Several of the hashtags used, such as #vapeordie and #swag, were seen as “cringey” and off-putting. Despite their own e-cigarette use, vapers and dual users were also extremely put-off by post elements referencing their visions of stereotypical vaping culture (Table 4: Q5.1). Non–tobacco users and smokers both suggested that the posts were trying too hard to be appealing and that vaping was less cool than smoking (Table 4: Q5.2).

Negative responses to marketing elements drawing on vaping culture were grounded in a preexisting, extremely negative view of stereotypical vapers. Across the smoking statuses, stereotypical vapers were commonly made fun of as “douches,” “Chads,” “neck-beards,” and “bros.” Several smokers and non–tobacco users suggested that vaping was inherently less cool than smoking. Participants from all smoking statuses were reluctant to be associated with vaping culture. Vapers and dual users spoke of trying to disassociate themselves from the culture depicted in the posts, while smokers were concerned that vaping would automatically lump them into the vaping culture. One smoker felt that the post sent a message that “if you vape, you will become like this.” The poor perception of vaping culture appeared to serve as a deterrent for some dual users and smokers to switch entirely to vaping (Table 4: Q5.3).

#### Marketing Should be Sensitive to Nicotine Addiction

Across all smoking statuses, several participants were upset about the ways in which post creators failed to consider the seriousness of nicotine addiction. Many users recognized that youth vaping was an issue and several complained that posts and e-liquids contained elements, such as sweet flavors and cartoons, that might be too appealing to younger users*.* Some of these concerns were grounded in concern for youth, while others were concerned that it made the vaping community look bad (Table 4: Q6.1). Two common concerns specific to nicotine users were that posts trivialized addiction and that they failed to promote products in a way that met the needs of those with nicotine addictions (Table 4: Q6.2). Nicotine users were generally accepting of their own addictions but expressed significant concern about the broader ramifications of marketing strategies that targeted children or would lead to continued addiction. Hashtags such as #vapeaddict created particularly negative responses (Table 4: Q6.3).

#### Warning Statements Reduce Post Appeal

Several participants across smoking statuses felt that the FDA-required warning statements about nicotine being addictive reduced post appeal. However, the impact on risk perception and appeal of the products promoted in posts was more ambiguous. Primarily, the warnings were seen as diminishing appeal because they were so large that they ruined the aesthetics and visual design of the post (Table 4: Q7.1). Some questioned the judgement of the Instagram user for applying such large and frequent warnings, not realizing that they were mandated. Other participants were aware of the requirement but were unclear about when the statements were required, particularly regarding sponsored users and e-liquids without nicotine. At the same time that they expressed dislike over the visual impact of the warnings, many nicotine users felt that the presence of the warnings was important to deter youth and novel users, although they were not always sure that the warnings would actually work to deter anyone.

Regarding personal risk perception, nicotine users were largely nonplussed by the warnings about nicotine addiction due to their current nicotine addictions and product familiarity (Table 4: Q7.2, Q7.3). Others felt that warning statements were so omnipresent in their lives, particularly on foods, that they tuned them out altogether. However, a few nicotine users did explicitly suggest that it was an unwelcome reminder of the potential harms of their vaping. For non–tobacco users, the warning statements had a clearer impact on risk perception and reduced product appeal. Many saw themselves as non–nicotine users, as reflected in the higher appeal of zero milligram nicotine e-liquids. Accordingly, the presence of the nicotine warnings reduced product appeal, and products in posts without warnings were often seen as safer. One non–tobacco user explained that the warnings were like a “road closed do not go here sign.”

## Discussion

### Primary Findings

The findings from this focus group study suggest that Instagram posts promoting e-liquids may hold appeal for individuals across smoking statuses, including non–tobacco users. The primary elements that create appeal are the trustworthiness of the Instagram account, the use of attractive design and flavor visuals, and the promotion of flavors and nicotine levels that meet personal preferences. The importance of visuals, particularly those depicting flavors, suggests that Instagram posts are well positioned for highlighting the often detailed and vivid art on e-liquid bottles. By contrast, post appeal is reduced by references to vaping subcultures, themes and hashtags that suggest post creators do not take nicotine addiction seriously, and the use of FDA-mandated warning statements. In short, participants weighed the account creator, the post content, and the product being promoted in making their assessment of posts. Each of these represent an area for potential regulatory action.

The elevated appeal and sense of authenticity created by sponsored users is of concern since Instagram is home to large volumes of posts made by users who indicate a sponsor or affiliate relationship with e-liquid and e-cigarette brands [3]. Prior Instagram research also supports that ads that resemble user-generated posts are considered more authentic and appealing than those that resemble traditional advertising [14,24]. Given the spread and appeal of sponsored promotions for e-liquids, this represents an important area for future research and action by both the FDA and the US Federal Trade Commission.

While findings suggest that limiting flavor options would likely help reduce post appeal among nonsmokers, it would likely have a similar effect on smokers and dual users who may, in the absence of full cessation, benefit from a full transition to e-cigarette use. Several prior studies have also suggested that non–tobacco flavors may play a role in smoking cessation for young adults [25,26]. Regulating the use of colors and cute/cartoon visuals on labels, which were a significant driver of initial appeal and a source of concern for some nicotine users, may be a more pragmatic approach. Further research should explore the viability of limiting appeal specifically among non–tobacco users. FDA-mandated warning statements on images do appear to have a promising effect on reducing the appeal of posts. Attractive and platform normative visuals play an essential role to establishing marketing appeal on Instagram [13,14], and it appears that large warnings may disrupt the positive effects of an otherwise good visual. This impact is distinct from the actual risk messaging of the warning, which had a more limited negative effect that was largely limited to nonsmokers. Echoing findings from Guillory et al [15], the actual content of the warnings may not be effective for nicotine users given that they already experience nicotine addiction. Further, regulators should ensure that the warning mandate does not inadvertently lead to more posts marketing nicotine-free e-liquids to avoid applying the warnings. Focus group findings suggest that an increase in marketing for zero milligram e-liquids may cause increased interest from non–nicotine users, while having minimal effects on e-cigarette users.

Finally, references to vaping subculture appeared to be the element that most strongly reduced the appeal of posts among participants. While early studies suggested that young adults found vaping to be “cool” [27,28], more recent work suggests that vaping has shifted into a stigmatized “uncool” behavior [29-32]. In this case, it appears that one of the most unappealing things an e-liquid post can do is prominently feature an image of a person who is seen as a stereotypical vaper. This is a particularly notable finding given earlier research suggesting that images of a person using an e-cigarette created appeal among smokers [33,34]. Both the drivers of this shift and the implications for e-cigarette uptake need further study. The health equity risks of stigmatizing e-cigarette users in a similar fashion to smokers should also be considered [35].

### Limitations

This study has several limitations. Although focus group participants were recruited from multiple social media platforms and offline settings, they may not be representative of all young adults. The overall number of smokers enrolled was also smaller than other status groups due to a low prevalence of smokers among those who contacted the study team to enroll. Additionally, smoking status was self-reported, and some participants may have been placed in focus groups not reflecting their true smoking status. The use of real Instagram posts and brands prevented a narrow focus on the impact of individual post elements in isolation, but significantly strengthened ecological validity.

### Conclusion

This research suggests that young adults consider a broad range of elements in assessing the appeal of e-liquid marketing on Instagram. Although flavors are important for ultimate appeal, participants also mentioned factors such as visual design, nicotine levels, and account trustworthiness as critical. The intersection between Instagram, as a visual platform, and the often-elaborate art on e-liquid bottles is particularly notable for those seeking to reduce the appeal of e-liquid marketing. For young adults who did not currently use tobacco, posts featuring attractive e-liquid designs in multiple flavors and zero milligrams of nicotine were particularly appealing and therefore of concern. Warnings reduced the visual appeal of posts among participants, although the specific phrasing mandated by the FDA may have limited impact on those who already face addictions to nicotine. Overall, the identification of nuances in post perceptions across smoking status groups suggests that it may be possible to target regulatory approaches specifically toward minimizing vaping appeal among non–tobacco users.

## Appendix

Multimedia Appendix 1 Summary of posts used to guide focus group discussion.

## Abbreviations

e-cigarettes: electronic cigarettes
e-liquids: electronic cigarette refill liquids
FDA: United States Food and Drug Administration
