# Supplementary material for: Assessing the Appeal of Instagram Electronic Cigarette Refill Liquid Promotions and Warnings Among Young Adults: Mixed Methods Focus Group Study
Source: J Med Internet Res. 2019 Nov 25;21(11):e15441. doi: 10.2196/15441 (PMC6902130; doi:10.2196/15441)
Supplement: Multimedia Appendix 1 [file jmir_v21i11e15441_app1.docx]

| **Post Identifier^1^** | **Post description** | **Warning statement** | **Account type** |
| --- | --- | --- | --- |
| Post 1a | Two packages of e-liquid on a counter in a vape shop. Package imagery features 8-bit style art of video game monsters and fake blood on the top of the package. Caption describes two flavor options: dragon fruit and tangerine with or without menthol. | Not present | Brick and mortar vape shop |
| Post 1b | E-liquid bottle surrounded by an illustration of ice-cubes with watermelon in them. Caption describes the flavor as “ripe watermelon and fresh strawberries with an icy finish.” | Present at top of image | Sponsored user mentioning affiliate status in user-info |
| Post 2a | Two identical e-liquid bottles on a windowsill. The label features black and white illustration of a man giving a presentation. Caption describes the flavor as “a perfect blend of pineapple, mango, kiwi, and the perfect amount of orange.” The bottle is labeled as 3mg of nicotine. | Not present, but warning label is visible on bottle | E-liquid brand |
| Post 2b | E-liquid bottle surrounded by colorful candy worms. Caption describes resemblance to “classic two toned gummy candies.” Visual and caption mention discount. | Present at top of image and a warning label is partially visible on bottle. | Online vape store |
| Post 3a/b | A “handcheck” with a man’s hand holding a mod e-cigarette and a bottle of e-liquid with a colorful illustration of a pie with Fruity Pebbles Cereal on it. The bottle is labeled as 0mg of nicotine. Caption gives name of mod device and states “PIE”. | Not present | Sponsored user with brands in user-info |
| Post 4a/b | A man in a baseball cap with multiple tattoos holds out an e-liquid bottle with an illustration of a flame on it while exhaling vapor. The caption reads, “rep what you love.” | Present at top of image | Personal account of an owner of an e-liquid brand, mentioned in user-info |
| Post 5a | Five colorful e-liquid bottles depicting candy flavors and candy visuals on labels. Caption and image describe sales pricing for bundles. | Not present | Online vape shop |
| Post 5b | An e-liquid bottle and package on a table outdoors. The e-liquid features a bare bottomed cartoon banana giving a thumbs up. The caption reads, “This flavor is B-A-N-A-N-A-S.” | Present at top of image | Online vape shop |

**Multimedia Appendix 1. Summary of Posts Used to Guide Focus Group Discussion**

^1^Posts labeled “a” were used in the first focus group for each smoking status. Posts labeled “b” were used in the remaining two focus groups for each smoking status.
